# Strength in numbers

**DOI:** 10.1038/s41467-020-17817-x

**Published:** 2020-09-09

## Abstract

The robustness of science is best revealed when independent investigations of the same problem arrive at similar conclusions. At *Nature Communications*, we commit to disregard from our editorial evaluation any competing works that are published while a submission to our journal is under review or under revision by the authors.

Being the first to publish a research result in a peer reviewed journal is prized in the current evaluation system for researchers. The increasingly popular practice of preprint posting shifts the attention to when a result is first made public, thus time-stamping the achievements of a research group independently of what others publish in the meantime. However, because preprints are not yet peer reviewed, this does not fully address the issues with the evaluation system.

Therefore, having your work ‘scooped’—that is, having a competing work published while yours is anywhere between a final draft and close to being accepted by a journal—can be disheartening and perceived to hinder proper credit attribution. In highly-competitive or fast-moving fields, priority of discovery is often seen as paramount (though it is worth mentioning that people can overestimate the long-term penalties of being second to the finish line).

We believe that journal policies can go a long way in mitigating the consequences of, as well as redefining the negative connotations associated with, being scooped. At *Nature Communications*, we want to help on both fronts, by implementing the below changes with immediate effect.

We believe that journal policies can go a long way in mitigating the consequences of, as well as redefining the negative connotations associated with, being scooped. At *Nature Communications*, we want to help on both fronts, by implementing the below changes with immediate effect.

In the first instance, we can help ensure that your results still get the recognition they deserve when a competing study is published while your manuscript is under revision. When we invite a revision of a manuscript following peer review, we commit to keep evaluating the work at subsequent review rounds on its own merits: in other words, although similar papers published by different groups in the meantime must be acknowledged and cited where appropriate, such publications will not constitute a reason to reject the revised manuscript.

In addition to our policy during review and revision, we will also adopt a more open stance when assessing new submissions whose results overlap considerably with a recently published paper. While each manuscript will still be judged on its own merits on a case-by-case basis, we will not consider the presence of a recent publication reporting similar results as a sole reason for declining publication of the work being evaluated, when it is clear that the two studies were carried out independently. The final decision on what constitutes a reasonable time-frame between publication of competing work and submission of a new study will be ultimately made by the editorial team. So, you should not be discouraged in submitting to *Nature Communications* if results similar to yours have just been reported elsewhere. Instead, we encourage you to discuss the related work in your cover letter.

After all, when two research groups carry out independent work leading to similar conclusions, they are in effect validating each other’s findings. The value of corroborating studies cannot be emphasised enough at a time when there is a widespread belief that science has a reproducibilityproblem. Similar results corroborated by different groups, through different analysis and experiments, increase confidence in the scientific endeavour and thereby enhance reproducibility efforts in the community. Furthermore, it is rare that two studies overlap entirely, so any new work will almost certainly report new information as well as supporting existing knowledge.

*Nature Communications* still aims to publish manuscripts that will be of high interest and have a significant impact in their respective fields, and our editorial standards are not changing. At the same time, we want to stimulate a move away from an excessive focus on novelty and the pressure to be ‘first’ in research, as this is not always indicative of the true value of the work. We hope that this change will be welcomed by our authors.

